# Effect of mangosteen peel extract as an antioxidant agent on the shear bond strength of orthodontic brackets bonded to bleached teeth

**DOI:** 10.1590/2177-6709.23.5.058-064.oar

**Published:** 2018

**Authors:** Ananto Ali Alhasyimi, Pinandi Sri Pudyani, Ikmal Hafizi

**Affiliations:** 1 Gadjah Mada University, Faculty of Dentistry, Department of Orthodontics (Yogyakarta, Indonesia).; 2 Gadjah Mada University, Faculty of Dentistry, Master Program of Biomaterial Science (Yogyakarta, Indonesia).

**Keywords:** Brackets, Bonding, Bleaching, Mangosteen peel extract

## Abstract

**Introduction:**

The number of patients who seek orthodontic treatment that may have a history of tooth bleaching is increasing over the time. Bleaching may influence the decrease of the bond strength of orthodontic brackets.

**Objective:**

To determine and prove the effect of mangosteen peel (MP) extract to reverse the reduced shear bond strength (SBS) of orthodontic brackets after bleaching.

**Methods:**

A total of 150 maxillary first premolar teeth were randomly divided into 6 experimental groups as follow (n=25): negative-control (N: no bleaching), positive-control (P: bleaching + no treatment), and the treatment groups (bleaching + 10% sodium ascorbate (SA), 10% (MP-10), 20% (MP-20) and 40% (MP-40) MP extract gel). After treatment, the brackets were bonded with the resin-modified glass ionomer cement, SBS testing was performed using universal testing machine, and the adhesive remnant index (ARI) was examined using stereoscopic microscope after debonding. The SBS data were analyzed by analysis of variance (Anova) and the Tukey test. For the ARI, the Kruskal-Wallis test was performed.

**Result:**

There was significant SBS difference (*p*< 0.001) between various groups. The group without bleaching showed significantly higher SBS (8.19 ± 2.26 MPa) compared to others, while SBS in the group treated with 40% MP gel was significantly higher (7.93 ± 1.92 MPa) than other groups treated with antioxidants. The failure of orthodontic brackets bonded after bleaching and treatment using MP extract occurred at the enamel-adhesive interface.

**Conclusion:**

The application of MP extract as an antioxidant after bleaching was effective in reversing the reduced shear bond strength of orthodontic brackets after bleaching.

## Resumo

**Introdução:**

o número de pacientes que procuram o tratamento ortodôntico e têm histórico de clareamento dentário tem aumentado. O clareamento pode levar à diminuição da resistência adesiva dos braquetes ortodônticos.

**Objetivos:**

comprovar a efetividade do extrato de casca de mangostão (CM) em reverter a diminuição da resistência ao cisalhamento de braquetes ortodônticos colados após o clareamento.

**Métodos:**

150 primeiros pré-molares superiores foram aleatoriamente divididos em seis grupos experimentais (*n*= 25): controle negativo (grupo N, sem clareamento), controle positivo (grupo P, clareamento + sem tratamento) e os grupos com tratamento (clareamento + ascorbato de sódio a 10% [grupo AS], gel de extrato de CM a 10% [grupo CM-10], a 20% [grupo CM-20] e a 40% [grupo CM-40]). Após o tratamento, os braquetes foram colados com cimento de ionômero de vidro modificado por resina e, depois, fez-se o teste de resistência ao cisalhamento (SBS) em uma máquina universal de ensaios. Após a descolagem dos braquetes, verificou-se o índice de adesivo remanescente (ARI), com o uso de um microscópio estereoscópico. Os dados da SBS foram submetidos a uma análise de variância (ANOVA) e ao teste de Tukey. Para o ARI, foi utilizado o teste de Kruskal-Wallis.

**Resultados:**

houve diferença significativa na SBS (*p*< 0,001) entre os diferentes grupos. O grupo sem clareamento mostrou resistência ao cisalhamento significativamente maior (8,19 ± 2,26 MPa) do que os outros grupos, enquanto a resistência ao cisalhamento do grupo tratado com o gel de extrato de CM a 40% foi significativamente maior (7,93 ± 1,92 MPa) do que nos outros grupos tratados com antioxidantes. A falha na colagem dos braquetes ortodônticos após o clareamento e tratamento com o extrato de CM ocorreu na interface adesivo/esmalte.

**Conclusão:**

a aplicação do extrato de CM como agente antioxidante foi efetiva em reverter a diminuição, que ocorre após o clareamento dentário, na resistência ao cisalhamento da colagem de braquetes ortodônticos.

## INTRODUCTION

Nowadays, tooth discoloration has become a big problem in all levels of the society. To overcome this problem, bleaching is the best treatment, since it does not involve a lot of dental structures when is performed, and provides a significant improvement on tooth appearance^1^ ^,^ ^2^. Bleaching using various whitening agents has been widely accepted by dentists and their patients as a tooth whitening method that is safe, simple and effective, with predictable result^3^.

An *in vitro* study showed that bleaching procedure is more effective and provides more significant results when performed before the use of orthodontic brackets rather than when it is done after debonding^4^. Unfortunately, bleaching may lead to a decrease in the bond strength of orthodontic brackets^5^ ^-^ ^7^. In-office bleaching may produce immediate results, but leaves more residual peroxide on the tooth surface, which might inhibit polymerization of the adhesive^8^. *In vitro* studies showed that application of synthetic antioxidants such as sodium ascorbate was effective in restoring the bond strength of brackets after bleaching^9^ ^-^ ^11^. 

The use of synthetic antioxidants may cause some health problems in human.^12^ Moreover, the efficiency level of synthetic antioxidants is lower than natural antioxidants, which has become the lessening factor of synthetic antioxidants use^13^. Mangosteen is a tropical fruit whose peels are sources of powerful natural antioxidants. Previous *in vitro* study proved that mangosteen peel extract is efficient in controlling oxidation reaction of free radical molecules, comparable to some commercial antioxidants^14^. Thus, the aim of this study was to determine the effect of mangosteen peel extract on the shear bond strength of orthodontic brackets to bleached teeth. The hypothesis to be tested was that mangosteen peel extract would reverse the reduced shear bond strength of brackets in bleached teeth.

## MATERIAL AND METHODS

### Preparation of specimens

**Figure 1:**
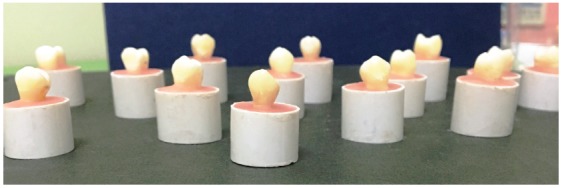
Specimens mounted in self-curing acrylic resin.

This study was approved by the Ethics Committee of Gadjah Mada University (Faculty of Dentistry, #00669/KKEP/FKG-UGM/EC/2016). One hundred and fifty maxillary first premolars (*n*= 150) recently extracted for orthodontic reasons with no defects, cracks, or restorations were chosen. After selection, the teeth were submitted to manual scaling with a periodontal curette, to remove organic debris. The teeth used for the study were cleaned and then decontaminated with a 0.5% chlorine solution for one week at room temperature, which was changed every other day, to prevent bacterial contamination. Each tooth was mounted in self-curing acrylic up to the cemento-enamel junction, with its long axis vertical (Fig 1). Specimens were then randomly divided into six groups (*n*= 25), as follow: Group 1 (negative control, N), unbleached teeth; Group 2 (positive control, P), bleached teeth with no treatment before bracket bonding; Group 3 (SA), bleached teeth, treated with 10% sodium ascorbate before bracket bonding; and Groups 4 (MP-10), 5 (MP-20) and 6 (MP-40), bleached teeth treated, respectively, with 10%, 20% and 40% mangosteen peel extract gel before bracket bonding (experimental groups). 

### Bleaching procedure

All of the specimens were bleached using 40% hydrogen peroxide (Opalescence® Boost, Ultradent, USA) on the enamel surfaces according to the manufacturer’s instructions. The thickness of the bleaching agent that was applied on each tooth’s enamel surface was 0.5 - 1 mm. After 20 minutes, it was washed with distilled water and dried using air syringe for 30 s. The bleaching procedure was applied twice, as recommended by the manufacturer’s instruction manual, for optimal results. 

### Application of the antioxidant

**Figure 2:**
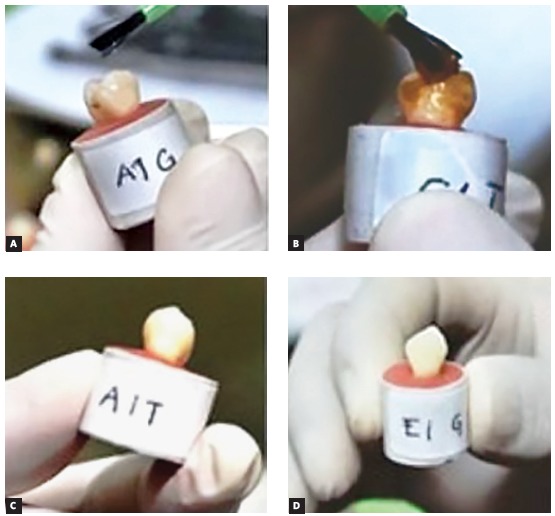
Specimens before application of SA (A); before application of MP (B); after application of SA (C); after application of MP (D). (SA = sodium ascorbate; MP = mangosteen peel).

Groups 3, 4, 5 and 6 were treated as follows: 10% sodium ascorbate gel (manufactured by *Laboratorium Penelitian dan Pengujian Terpadu*, LPPT, Indonesia) and 10%, 20%, and 40% mangosteen peel gel (manufactured with mangosteen peel extract, CMC-Na 2%, glycerin, propylene glycol, propylparaben and methylparaben, by LPPT, Indonesia). Antioxidant was applied 0.5 - 1 mm onto the enamel surfaces of the embedded teeth and agitated for 30 s with a sterile brush following the bleaching process (Fig 2). After 10 minutes, it was washed with distilled water and gently dried by air jets. After completion of the procedure, the samples were stored in artificial saliva solution for 24 hours prior to bonding.

### Bonding of brackets

One hundred and fifty stainless steel pre-adjusted Edgewise upper bicuspid brackets (American Orthodontics, USA), with 0.022-in slot and micro-etched base with a surface area of 10.64 mm^2^, were used for the study. The brackets were handled at all times with bonding tweezers, to avoid contamination. The enamel surface was conditioned with 10% polyacrylic acid (GC Corporation, Tokyo, Japan), then the brackets were bonded with a resin-modified glass ionomer cement (Fuji Ortho Light Cure, GC Corporation, Tokyo, Japan), according to the manufacturer’s instructions. After mixing the powder and liquid, the homogenized mixture was spread on the bracket’s base. A bracket positioning gauge was used to place the brackets on the mid-buccal surfaces of the teeth at least 4 mm away from the buccal cusp ridges. Meanwhile, the bracket slot was perpendicular to the tooth coronal long axis. Furthermore, each bracket was light-cured for 40 seconds according to the manufacturer (10 seconds per side: occlusal, cervical, mesial, and distal) using a quartz-tungsten-halogen (QTH) light-curing unit (Litex 680A, Dentamerica, USA) with a light intensity of 450 mW/cm^2^. Based on the *in vitro* bond strength study by Henkin et al,^15^ in the present study, all specimens were stored in distilled water at 37°C for 24 hours after bonding, prior to SBS analysis.

### Shear bond strength analysis

**Figure 3:**
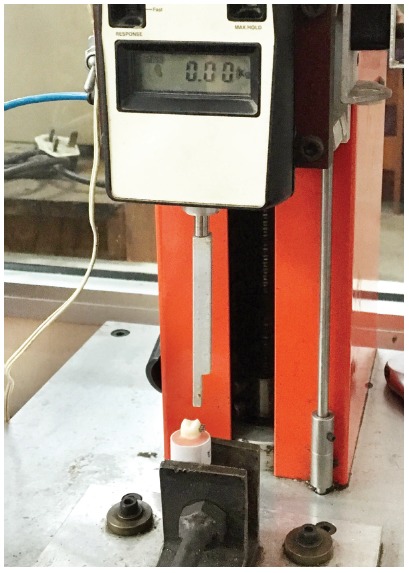
Universal testing machine used for determining the shear bond strength.

The shear bond strength (SBS) test was performed in a universal testing machine (Pearson Panke Equipment Ltd., London), with a 50 kg load cell, at a speed of 1.0 mm/min until the removal of the brackets. The chisel was positioned parallel to the surface of the tooth/bracket interface, to allow force transmission in the occluso-gingival direction (Fig 3). The results obtained were converted to megapascals (MPa) by dividing the debonding force (in Newton) by the bracket base area (10.64 mm^2^). Immediately after bracket debonding, the enamel surface of each specimen was examined under 10× magnification with a stereoscopic microscope (Nikon, SMZ-2T, Japan), to determine the amount of residual adhesive. Adhesive remnant index (ARI) scores at the failure sites were recorded according to the classification of Artun and Bergland,^16^ as follows: score 0, no adhesive left on the tooth; score 1, less than half of the adhesive left on the tooth; score 2, more than half of the adhesive left on the tooth; and score 3, all of the adhesive left on the tooth.

### Statistical analysis

The shear bond strength data of the groups were subjected to a test of normality and homogeneity. With respect to this, Analysis of Variance (Anova) was used to determine the significance between the groups. The significance level for all statistical tests was set at *p*< 0.05, and Tukey’s HSD *post-hoc* test was used to detect pairwise differences between the groups (Table 1). The ARI scores were evaluated by Kruskal-Wallis analysis. To determine the differences between the groups, a Mann-Whitney U test was performed. Statistical analysis was processed with the SPSS 21.0 software (SPSS Inc., Chicago, Illinois, USA).

**Table 1:** Descriptive statistics and results of the ANOVA and Tukey tests comparing the shear bond strengths in the six groups tested.

Group	n	SBS (MPa)	Sig*	p-value				
				P	SA	MP-10	MP-20	MP-40
N	15	8.19 ± 2.26	p=0.000^a^	0.000^a^	0.007^a^	0.087^b^	0.363^b^	0.998^b^
P	15	4.57 ± 1.49			0.052^b^	0.004^a^	0.000^a^	0.000^a^
SA	15	6.19 ± 1.82				0.955^b^	0.627^b^	0.029^a^
MP-10	15	6.68 ± 2.06					0.982^b^	0.234^b^
MP-20	15	7.07 ± 2.29						0.65^b^
MP-40	15	7.93 ± 1.92						

Values are presented as mean ± standard deviation or p-value only. *ANOVA, ^a^Significant differences between groups (*p*< 0.05). ^b^No significant differences between groups (*p*> 0.05). N = negative-control; P = positive-control; SA = sodium ascorbate; MP-10 = 10% mangosteen peel extract; MP-20 = 20% mangosteen peel extract; MP-40 = 40% mangosteen peel extract. ANOVA = Analysis of variance; SBS = shear bond strength.

## RESULTS 

Table 1 contains the mean and standard deviation for the SBS of specimens in the six study groups. These descriptive statistics clearly indicate the variation in SBS among the six groups, with the higher bond strength present in the negative-control group (8.19 ± 2.26 MPa), and the lower in the positive-control group (4.57 ± 1.49 MPa). It can be observed that Groups 3, 4, 5 and 6, which were subjected to antioxidants treatment after bleaching, showed an improvement in SBS, compared to the positive-control group, while the group treated with MP-40 showed the highest SBS (7.93 ± 1.92 MPa), compared to other groups treated with antioxidants.

The results of the ANOVA indicated statistically significant differences among the tested groups (*p*= 0.000). The Tukey test showed that the SBS of Group 1 (negative-control) was significantly higher than other groups. Furthermore, no statistically significant difference in SBS value was found between Group 1 and groups treated with 10%, 20%, and 40% MP extract (*p*> 0.05).

The ARI scores for all the tested groups are listed in Table 2. The results of the Kruskal-Wallis test showed significant differences among the groups (*p*= 0,012). ARI scores equal to 0 and 1 were more prevalent, while ARI scores equal to 2 and 3 were less prevalent.

**Table 2:** Frequency distribution of the ARI scores of the groups and results of the Kruskal-Wallis test.

Group	ARI scores								Kruskal-Walis	
	0		1		2		3		Chi-square	p
	n	%	n	%	n	%	n	%		
N	9	-36	16	-64	0	0	0	0	8.096	0,012*
P	6	-24	11	-44	6	-24	2	-8		
SA	7	-28	12	-48	5	-20	1	-4		
MP10	8	-32	13	-52	3	-12	1	-4		
MP20	7	-28	18	-72	0	0	0	0		
MP40	6	-24	19	-76	0	0	0	0		

*ARI scores: 0 = no adhesive on the tooth; 1 = less than half of the adhesive left on the tooth surface; 2 = half of the adhesive or more left on tooth surface; 3 = all adhesive left on tooth surface, failure between adhesive and bracket base. * Significant differences between groups (*p*< 0.05). ARI = Adhesive remnant index.

## DISCUSSION

The results, in general, showed there were increases in SBS in groups with antioxidant treatments. Group 1 showed the highest SBS values; conversely, Group 2 showed the lowest SBS values. The reduced SBS in Group 2, compared to other groups, may be due to the residual oxygen layer remaining after the bleaching process, which could have interfered with the resin infiltration into etched enamel and inhibit polymerization of resins that cure via a free-radical mechanism^17^. Reynolds^18^ recommended that the suitable minimum bond strength for clinical requirements, being able to resist masticatory and orthodontic forces, ranges from 6 to 8 MPa. The groups treated with antioxidant showed mean values ranging from 6 to 8 MPa, confirming that even samples subjected to peroxide agents could withstand the stresses from orthodontic forces.

Groups with antioxidants treatments showed significantly higher bond strength than Group 2 (positive-control). These findings are in accordance with the fact that the use of antioxidants immediately following bleaching could neutralize the residual oxygen and reverse the reduced bond strength^19^. Studies have shown that the use of antioxidants can definitely overcome the inclusion of peroxide ions. An antioxidant solution of 10% sodium ascorbate (SA) applied on the bleached enamel surface for 10 minutes effectively restored the reduced bond strength. Sodium ascorbate is a potent antioxidant, capable of removing the reactive free radicals and neutralizing their effect. The mechanism by which sodium ascorbate reverses the reduction in bond strength is that sodium ascorbate allows free-radical polymerization of the adhesive resin to proceed and avoids early termination, by repairing the altered redox potential of the oxidized bonding substrate, and hence neutralizes the compromised bonding^20^. In the process subsequent to the application of sodium ascorbate, SBS values reached a level almost similar to the MP extract groups. However, groups with 40% MP extract treatment showed significantly higher bond strength than the group with SA treatment, which could be attributed to the fact that the antioxidant present in MP extract is more potent than the SA.

Group 1 (unbleached teeth) showed significantly highest bond strength than the other groups. Previous studies have shown that when teeth are exposed to bleaching agents, there is a change in the structure of enamel and the bond strength. The bleaching procedure may decrease the microhardness and weaken the mechanical properties of the tooth, and thus reduce the bond strength of brackets to tooth surface. Another possible reason is attributed to the fact that the bleaching agent also affects the collagen network of dentin, resulting in denaturation and relative instability of the dentin organic matrix, therefore decreasing bond strength^5^ ^-^ ^9^. Furthermore, the results showed no significant difference in SBS values between Group 1 (unbleached teeth) and the groups treated with 10%, 20% and 40% MP extract (*p*> 0.05). In addition, SBS values of the group with 40% MP extract treatment reached a level almost similar to the negative-control group. It is indicated that the treatment with MP extract may approximate the bond strength values of teeth without the bleaching procedure. 

Application of MP extract on enamel bleached with 40% hydrogen peroxide neutralized the effect of residual oxygen molecules on the bleached enamel surface and increased the SBS of orthodontic brackets. Mangosteen (*Garcinia mangostana L.*) peel presented potential antioxidant properties, and it was stronger when compared to its pericarp and leaves as well^21^. Antioxidants are substances that can delay or prevent the oxidation process by inhibiting the initiation or propagation of oxidation chain reaction. Suttirak and Manurakchinakorn^13^ showed that the antioxidant properties of MP extract can inhibit or delay oxidation by scavenging free radicals (i.e. reactive oxygen species, e.g. hydroxyl, superoxide, nitric oxide, thiyl and peroxyl) by donating a hydrogen atom or electron, which convert their radicals to the more stable products. 

Alpha(α)-Mangostin was the first xanthone isolated from MP and has been reported as an antioxidant by scavenging, in a singlet oxygen concentration-dependent way^22^ ^,^ ^23^. Furthermore, MP extract contains epicatechin as the predominant monomeric unit, reported as a better free radical scavenger^24^.

The ARI scores indicated significant differences among the various groups, although ARI scores of 0 and 1 were seen with higher frequency. In MP 20 and MP 40 groups, there was a higher frequency of ARI scores equal to 1. This means that failures occurred at the enamel-adhesive interface. This could be clinically advantageous since, when brackets fail at the enamel-adhesive interface, the less residual adhesive remains, and tooth clean-up is likely to be easier and faster^25^ ^-^ ^27^. Treatment success in clinical orthodontics is stated upon the easy and efficient removal of remaining adhesive without iatrogenic damage to the underlying enamel when the attachments are debonded^28^. The use of a bur or sandblaster to clean the remaining adhesive from the tooth surfaces can also lead to surface scratches, cracking, loss of sound enamel and may increase chairside time. ^25^ ^,^ ^29^

Extensive prior studies have also noted that delayed bonding after bleaching can effectively restore the shear bond strength of brackets, since the decrease in bond strength to freshly bleached enamel has been shown to be temporary. The recommended post-bleaching time for bonding procedures ranges from one to three weeks^10^ ^,^ ^26^ ^,^ ^30^ ^,^ ^31^. Previous studies^10^ ^,^ ^30^ reported that waiting for seven days before bonding orthodontic bracket was sufficient to ensure adequate bond strength after bleaching. A study by Machado et al^31^ has shown that intervals of more than 15 days are required to restore the adhesive properties of enamel prior to bonding any orthodontic appliances. In another study, Nascimento et al^26^ found that delaying bracket bonding for three weeks is required to attain acceptable bond strength. On the other hand, once patients have their teeth bleached, they often become immediately interested in orthodontic treatment.^10^ Thus, the presence of an antioxidant may be an alternative clinical option to having to wait, and more importantly, may reduce the necessity of postponing bracket bonding.

The present study used resin-modified glass ionomer cement (RMGIC) to bond the brackets because this material has become popular among orthodontists due to its advantages over composite resins, such as its appropriate results on SBS in areas difficult to isolate from a wet environment, releasing fluoride and effectively preventing white spot lesions.^32^ It seems important to assess the behavior of RMGIC applied to the bleached teeth, and also to evaluate possible surface treatments to achieve the best outcome, considering its broad use. RMGIC seemed to be less sensitive to bleaching because the oxygen inhibits the polymerization of the composite resin. However, obviously, the low fraction of 2-hydroxyethylmethacrylate (HEMA) molecules in RMGIC may make it vulnerable to oxygen^33^. In addition, the application of antioxidants treatment increased the SBS eventually, showing that free oxygen radicals did have a considerable negative impact on the mean SBS and inhibit the polymerization of RMGIC.

## CONCLUSION

Within the limitations of the present study, the application of mangosteen peel extract as an antioxidant was effective in increasing the SBS of brackets in bleached teeth. However, additional clinical and laboratory studies are required before mangosteen peel extract can be used in daily clinical practice.
